# Supplementary material for: Evidence-Based Nutritional Recommendations for Maintaining or Restoring Nutritional Status in Patients with Amyotrophic Lateral Sclerosis: A Systematic Review
Source: Nutrients. 2025 Feb 24;17(5):782. doi: 10.3390/nu17050782 (PMC11901627; doi:10.3390/nu17050782)
Supplement: Supplementary file 1 [file nutrients-17-00782-s001.zip › Supplementary File S2. Search strategies in databases.pdf]

Supplementary File S2. Search strategies in databases.

Database	Equations
PubMed/MEDLINE	"amyotrophic lateral sclerosis" OR "motor neuron disease" AND "practice guideline"
Embase	(amyotrophic AND lateral AND sclerosis OR 'motor neuron disease') AND 'nutrition' AND 'practice guideline'
Scopus	"amyotrophic lateral sclerosis" OR "motor neuron disease" AND "nutrition therapy"
SciELO	(((*amyotrophic lateral sclerosis) OR (motor neuron disease)) AND (nutrition therapy) OR (diet))
Web of Science	(((ALL=(amyotrophic lateral sclerosis)) OR ALL=(motor neuron disease)) AND ALL=(nutrition therapy)) AND ALL=(guideline)
LILACS	"amyotrophic lateral sclerosis" OR "motor neuron disease" AND "dietary supplements"
Science Direct	("amyotrophic lateral sclerosis" OR "motor neuron disease") AND ("nutrition therapy")
Google Scholar	"amyotrophic lateral sclerosis" OR "motor neuron disease" AND "nutrition therapy" AND "diet" AND "guideline"
