# Supplementary material for: Evidence-Based Nutritional Recommendations for Maintaining or Restoring Nutritional Status in Patients with Amyotrophic Lateral Sclerosis: A Systematic Review
Source: Nutrients. 2025 Feb 24;17(5):782. doi: 10.3390/nu17050782 (PMC11901627; doi:10.3390/nu17050782)
Supplement: Supplementary file 1 [file nutrients-17-00782-s001.zip › Supplementary File S3. AGREE II score sheet.pdf]

Supplementary File S3. AGREE II score sheet.

Domain	Item		References of studies and Guidelines*									
		27	29	30	31	32	33	35	36	37	39	40
			AGREE II Rating: 1 (Strongly Disagree) to 7 (Strongly Agree)									
Scope and purpose	1. The overall objective(s) of the guideline is (are) specifically described.	7	7	7	6	7	1	7	7	1	7	7
	2. The health question(s) covered by the guideline is (are) specifically described.	7	7	7	6	7	7	7	7	7	7	7
	3. The population (patients, public, etc.) to whom the guideline is meant to apply is specifically described.	7	7	7	6	7	7	7	7	6	7	7
Stakeholder involvement	4. The guideline development group includes individuals from all the relevant professional groups.	7	7	4	7	7	6	1	7	2	7	3
	5. The views and preferences of the target population (patients, public, etc.) have been sought.	3	5	1	1	7	7	1	2	1	7	7
	6. The target users of the guideline are clearly defined.	7	7	7	7	7	7	7	7	7	7	7
Rigor of development	7. Systematic methods were used to search for evidence.	7	7	7	5	6	7	7	7	2	7	7
	8. The criteria for selecting the evidence are clearly described.	7	7	6	4	7	7	7	7	1	7	7
	9. The strengths and limitations of the body of evidence are clearly described.	7	7	2	6	7	5	7	7	2	7	1
	10. The methods for formulating the recommendations are clearly described.	7	7	7	4	7	7	7	7	7	7	7
	11. The health benefits, side effects and risks have been considered in formulating the recommendations.	7	7	7	6	7	6	6	7	7	6	7

	12. There is an explicit link between the recommendations and the supporting evidence.	7	7	6	7	7	7	7	6	7	7	7
	13. The guideline has been externally reviewed by experts prior to its publication.	7	7	7	1	7	1	1	7	7	7	7
	14. A procedure for updating the guideline is provided.	1	7	1	1	1	1	2	6	2	1	1
Clarity of presentation	15. The recommendations are specific and unambiguous.	7	7	7	7	7	7	7	7	7	7	7
	16. The different options for management of the condition or health issue are clearly presented.	7	7	7	7	6	7	7	7	7	7	7
	17. Key recommendations are easily identifiable.	7	7	7	7	7	7	7	7	7	7	7
Applicability	18. The guideline describes facilitators and barriers to its application.	7	7	7	5	6	4	7	6	4	6	6
	19. The guideline provides advice and/or tools on how the recommendations can be put into practice.	7	7	5	7	7	4	7	7	4	5	7
	20. The potential resource implications of applying the recommendations have been considered.	5	4	4	7	4	2	1	6	1	5	1
	21. The guideline presents monitoring and/ or auditing criteria.	7	7	7	1	1	1	1	1	1	1	1
Editorial independence	22. The views of the funding body have not influenced the content of the guideline.	7	7	7	7	7	1	1	7	7	7	7
	23. Competing interests of guideline development group members have been recorded and addressed.	7	7	7	7	7	7	1	7	7	7	7
**Overall Guideline Assessment**	**Overall quality of the report based on a scale of 1 (lowest quality) to 7 (highest quality).**	**6**	**7**	**6**	**5**	**6**	**5**	**5**	**6**	**4**	**6**	**6**

*Reference of the studies and guidelines:

27. Burgos R, Bretón I, Cereda E, et al. ESPEN guideline clinical nutrition in neurology. Clin Nutr 2018;37(1):354-396.

29. Shoesmith C, Abrahao A, Benstead T, et al. Canadian best practice recommendations for the management of amyotrophic lateral sclerosis. CMAJ 2020;192(46):E1453-E1468.
30. Andersen PM, Abrahams S, Borasio GD, et al. EFNS guidelines on the clinical management of amyotrophic lateral sclerosis (MALS)-revised report of an EFNS task force. Eur J Neurol 2012 Mar;19(3):360-75.
31. Boostani R, Olfati N, Shamshiri H, Salimi Z, Fatehi F, Hedjazi SA, et al. Iranian clinical practice guideline for amyotrophic lateral sclerosis. Front Neurol. 2023;14:1154579.
32. Urushitani M, Warita H, Atsuta N, Izumi Y, Kano O, Shimizu T, et al. The clinical practice guideline for the management of amyotrophic lateral sclerosis in Japan-update 2023. Rinsho Shinkeigaku. 2024;64(4):252–71.
33. Van Damme P, Al-Chalabi A, Andersen PM, Chiò A, Couratier P, De Carvalho M, et al. European Academy of Neurology (EAN) guideline on the management of amyotrophic lateral sclerosis in collaboration with European Reference Network for Neuromuscular Diseases (ERN EURO-NMD). Euro J of Neurology. 2024;31(6):e16264. linical care of patients with amyotrophic lateral sclerosis. Lancet Neurol 2007;6(10):913-25.
35. Heffernan C, Jenkinson C, Holmes T, et al. Nutritional management in MND/ALS patients: an evidence based review. Amyotroph Lateral Scler Other Motor Neuron Disord 2004;5(2):72-83.
36. Miller RG, Jackson CE, Kasarskis EJ, et al. Practice parameter update: the care of the patient with amyotrophic lateral sclerosis: drug, nutritional, and respiratory therapies (an evidence-based review): report of the Quality Standards Subcommittee of the American Academy of Neurology. Neurology 2009;73:1218–26.
37. Petri S, Grehl T, Grosskreutz J, Hecht M, Hermann A, Jesse S, et al. Guideline "Motor neuron diseases" of the German Society of Neurology (Deutsche Gesellschaft für Neurologie). Neurol Res Prática. 2023;5(1):25.
39. Oliver D, Radunovic A, Allen A, McDermott C. The development of the UK National Institute of Health and Care Excellence evidence-based clinical guidelines on motor neurone disease. Amyotroph Lateral Scler Frontotemporal Degener 2017;18(5-6):313-323.
40. Brasil. Ministério da Saúde. Secretaria de Ciência, Tecnologia, Inovação e Insumos Estratégicos em Saúde. Departamento de Gestão e Incorporação de Tecnologias e Inovação em Saúde. Protocolo Clínico e Diretrizes Terapêuticas da Esclerose Lateral Amiotrófica [recurso eletrônico] 2021.
